# Triphala Suppresses Growth and Migration of Human Gastric Carcinoma Cells* In Vitro* and in a Zebrafish Xenograft Model

**DOI:** 10.1155/2018/7046927

**Published:** 2018-12-10

**Authors:** Jokyab Tsering, Xianda Hu

**Affiliations:** Beijing Tibetan Hospital, China Tibetology Research Center, Beijing, China

## Abstract

**Objectives:**

Triphala is an extensively prescribed traditional medicinal formula with potential therapeutic effects on various malignancies such as breast, colon, pancreas, prostate, ovarian, cervical, endometrial, and lymphatic cancer as well as melanoma. This study aimed to investigate Triphala for antitumor activities against gastric cancers.

**Methods:**

* In vitro* tumor growth and migration of human gastric cancer cells were examined using the CCK-8 and Transwell assays, respectively.* In vivo* tumor progression was studied in a zebrafish xenograft model. The anticancer activity of Triphala was quantified as growth and metastasis inhibition rate. The underlying molecular mechanism was investigated by Western blotting.

**Results:**

The CCK-8 and Transwell experiments indicated that Triphala significantly decreased tumor proliferation and suppressed cell migration* in vitro*. The zebrafish xenograft study revealed that administration of Triphala inhibited the xenograft growth and metastasis of transplanted carcinoma cells* in vivo*. Western blotting analysis demonstrated an inhibition of phosphorylation of EGFR, Akt, and ERK in the presence of Triphala, indicating that its antineoplastic mechanism is associated with the regulation of the EGFR/Akt/ERK signaling cascade.

**Conclusion:**

Triphala is a promising antineoplastic agent for the treatment of gastric carcinomas with significant antiproliferative and antimetastatic activities.

## 1. Introduction

Despite a significant decrease in both incidence and mortality over the past decades, gastric cancer remains a major global health burden as the fifth most frequently diagnosed malignancy and the third most common cause of death from cancer worldwide with a 5-year overall survival rate of less than 40% [1, 2]. Conventional therapeutic approaches for gastric cancer include surgical resection, radiotherapy, and chemotherapy. Complementary and alternative medical interventions are also frequently used as adjuvant therapies to improve treatment outcomes and reduce side effects [3].

Triphala, or* ‘Bras Bu gSum Thang* in Tibetan, which consists of three equal proportions of the fruits of* Terminalia chebula* Retz.,* Terminalia belerica* (Gaertn.) Roxb, and* Phyllanthus emblica* Linn., is a widely prescribed traditional remedy that has been clinically used for thousands of years as treatment of various diseases and disorders. Numerous publications indicate that Triphala exerts potent antineoplastic activities against different types of cancer, including breast, colon, pancreas, prostate, ovarian, cervical, endometrial, and lymphatic cancer as well as melanoma [4, 5]. However, whether Triphala has similar effects on gastric cancer has not yet assessed in relevant reports. In this study, we evaluated the effect of Triphala on gastric cancer* in vitro* and* in vivo*.

## 2. Materials and Methods

### 2.1. Preparation of Triphala Extract

Finely powdered Triphala (Dabur India Ltd., Alwar, India; batch number: AL1675) which contained equal amounts of* Terminalia chebula*,* Emblica officinalis,* and* Terminalia bellerica* was extracted with ultrapure water (substrate/extractant: 1/10 (w/v), reflux, 1 h). The precipitate was removed by centrifugation (2860 g, 15 min), followed by filtration through a 0.45-*μ*m membrane filter (Merck Millipore Ltd., Cork, Ireland). The filtrate solvent was removed by rotary evaporation followed by lyophilization. The yellowish green extract powder was weighed and stored at −20°C.

### 2.2. Cell Culture

Human gastric tumor cell line MGC-803 was maintained and provided by China Infrastructure of Cell Line Resource. The cells were propagated in high-glucose Dulbecco's modified Eagle's medium (H-DMEM) (Thermo Fisher Scientific Inc., Waltham, USA) supplemented with 10% fetal bovine serum (FBS) (Corning Inc., Corning, USA) and incubated at 37°C under 5% CO_2_ atmosphere.

### 2.3. *In Vitro* Cell Growth Assay

Gastric tumor cells were seeded in 96-well plates at a density of 1 × 10^4^ cells per well for 24 h for attachment and then cultured with increased concentrations of drugs (0, 50, 100, and 150 *μ*g/mL) for 48 h. Viability of MGC-803 cells was determined using the standard cell counting kit- (CCK-) 8 assay according to the protocol recommended by the manufacturer (Dojindo Inc., Kumamoto, Japan). Optical density (OD) was measured at 450 nm with a microplate reader (Beijing Pulang New Technology Co., Beijing, China), and the inhibition rate was calculated as follows: Growth inhibition rate (%) = (1 - Absorbance of experimental group / Absorbance of control group) × 100%. IC_50_ values were calculated by probit analysis using SPSS Statistics 19.0 software (IBM, Chicago, USA). The cytotoxic activity of Triphala was visualized using Calcein-AM/Propidium Iodide (PI) fluorescence probes according to the manufacturer's instructions (Dojindo), followed by observation and photography with a fluorescence microscope (Leica Microsystems GmbH, Wetzlar, Germany).

### 2.4. *In Vitro* Cell Migration Assay

Analysis of the migratory capability of gastric cells was performed with the Transwell Boyden chamber assay. Cells in the logarithmic growth phase were collected and resuspended with serum-free H-DMEM medium and seeded in the upper chamber of 24-well Transwell plates (Corning) at a density of 5 × 10^4^ cells per well, while regular medium containing H-DMEM and 10% FBS was introduced into to the lower chamber. After 24 h of incubation at 37°C, cells remaining in the upper side of the permeable membrane were wiped off using cotton swabs, while cells migrating to the bottom wells through the membrane micropores were fixed and stained using 0.1% crystal violet. The OD values were measured at 630 nm with a microplate reader, and the inhibition rate was calculated as follows: Migration inhibition rate (%) = (1 - Absorbance of experimental group / Absorbance of control group) × 100%.

### 2.5. Zebrafish Embryos

Wild type AB strain of zebrafish (*Danio rerio*) was obtained from Hangzhou Hunter Biotechnology Co., Ltd., Hangzhou, China. Adult zebrafishes were maintained under standard laboratory conditions, and embryos were generated by natural pairwise mating [6]. The embryos of 2 days postfertilization (dpf) were anesthetized with tricaine (Sigma-Aldrich Corp., St Louis, USA) and positioned on a Petri dish for microinjections.

### 2.6. *In Vivo* Antitumor Assay

The human gastric carcinoma cells were resuspended in phosphate buffer solution (PBS) and incubated with CM-Dil (Thermofisher) at final concentration of 1 *μ*g/L for 4 min at 37°C followed by an additional 15 min at 4°C. Fluorescence-labeled cells were then loaded into capillary needles and injected into the abdominal perivitelline space of zebrafish embryos with a nanoliter injector (Narishige, Tokyo, Japan). After injection, the tumor-bearing embryos were transferred into a 24-well plate and acclimated in embryo water at 35°C for 24 h and then incubated at 0, 50, 100, and 150 *μ*g/mL Triphala extract for 48 h. The tumor growth and metastasis were observed and imaged using fluorescence inverted microscope (Nikon Inc., Tokyo, Japan). The mean fluorescence intensity (MFI) was analyzed using ImageJ software (National Institutes of Health, Bethesda, USA). The growth inhibition rate was calculated as follows: inhibition rate (%) = (1 - MFI of experimental group / MFI of control group) × 100%. The* in vivo* migratory inhibition activity of Triphala was expressed as occurrence rate of metastasis. The metastasis inhibition rate was calculated as follows: inhibition rate (%) = (1 – incidence of experimental group / incidence of control group) × 100%.

### 2.7. Western Blot Assay

MGC803 cells were cultured in 10 cm Petri dishes and grown to approximately half confluence and then treated with 0, 50, 100, and 150 *μ*g/mL Triphala extract for 48 h. Cells were collected and lysed in RIPA buffer supplemented with a protease and a phosphatase inhibitor cocktail (Sigma-Aldrich Corp., USA). Cell lysates were separated by SDS-PAGE and transferred to a nitrocellulose membrane, which was probed with p-EGFR, p-ERK1/2, p-AKT monoclonal antibodies (Cell Signaling Technology, Danvers, USA), and subsequently incubated with secondary antibodies (Abcam, USA) for detection using enhanced chemiluminescence (ECL) according to the manufacturer's instructions (Thermo Fisher).

### 2.8. Statistical Analysis

Data are presented as the means ± standard deviations (SD) of triplicated independent experiments. Statistical analyses were performed by one-way ANOVA with the least significant difference (LSD) post hoc multiple comparison tests using SPSS Statistics 19.0 software (IBM, Chicago, USA). P value of <0.05 was considered as statistically significant.

## 3. Results

### 3.1. Triphala Inhibited Proliferation and Migration of Gastric Tumor Cells* In Vitro*

**Figure 1:**
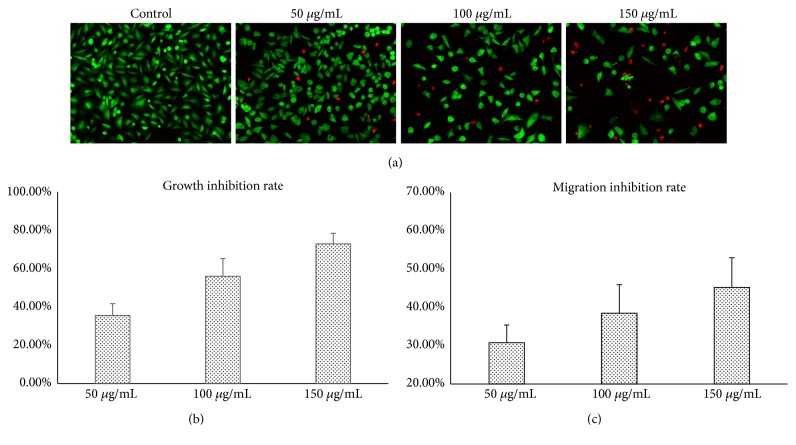
Assessment of the* in vitro* antitumor effects of Triphala on the human gastric carcinoma cell line MGC803. (a) Representative images of randomly selected microscopic fields of gastric tumor cells stained with Calcein-AM/PI after 48 h treatment with increasing concentrations (0, 50, 100, and 150 *μ*g/mL) of Triphala. (b) Growth inhibition rates of tumor cells after 48 h exposure to Triphala, which were determined using quantitative CCK-8 assay. (c) Migration inhibition rates of tumor cells administrated with Triphala, which were examined using the Transwell assay.

The* in vitro* antitumor and antimigratory properties of Triphala were determined by CCK-8 and Transwell assay, respectively. The quantitative CCK-8 assay demonstrated a dose-dependent growth inhibition of human gastric tumor MGC-803 cells with a half maximal inhibitory concentration (IC_50_) value of 86.08±3.87 *μ*g/mL (Figure 1(b)). The result was confirmed by the live/dead cell staining assay, which clearly demonstrated that an increase of the Triphala concentration was associated with a decrease of the Calcein-AM stained cell population and an increase in the number of PI-labeled nuclei (Figure 1(a)). Furthermore, the Transwell migration assay revealed a significant negative correlation between the concentration of Triphala and the vertical migration capacity of MGC-803 cells (Figure 1(c)).

### 3.2. Triphala Inhibited Tumor Growth and Metastasis in the Zebrafish Xenograft Model

**Figure 2:**
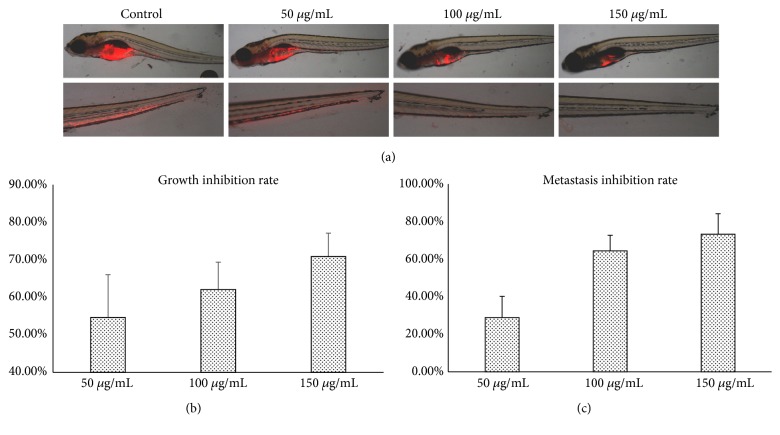
Evaluation of the* in vivo* antitumor effects of Triphala in zebrafish xenografted with the human gastric carcinoma cell line MGC803. (a) Representative photographs of the body (upper panels) and tail (lower panels) area of tumor-bearing zebrafishes exposed to increasing concentration (0, 50, 100, and 150 *μ*g/mL) of Triphala for 48 h. The* in vivo* growth and metastasis are indicated by red stained tumor cells detected with the fluorescence microscope. The growth inhibition rates (b) and metastasis inhibition rates (c) were calculated as percentages relative to the control values.

The* in vivo* antineoplastic and antimetastatic activities of Triphala were evaluated in a xenograft model of human gastric tumor MGC-803 cells. Fluorescence-labeled tumor cells were localized by fluorescence imaging and fluorescence intensities were processed with image analysis software. As compared with the fluorescence intensity of the control group, Triphala-treated fish bodies had a declining fluorescence intensity that positively correlated with the increasing Triphala dose (Figures 2(a) and 2(b)). Meanwhile, decreasing metastatic incidences in tumor-bearing zebrafishes were detected with rising concentration of Triphala (Figures 2(a) and 2(c)). These findings indicated that Triphala is capable of mediating tumor growth and metastasis in a dose-dependent manner* in vivo*, which are consistent with the* in vitro* studies.

### 3.3. The Antitumor Effects of Triphala Are Associated with the Regulation of the Epidermal Growth Factor Receptor (EGFR) Signaling Cascade

**Figure 3:**
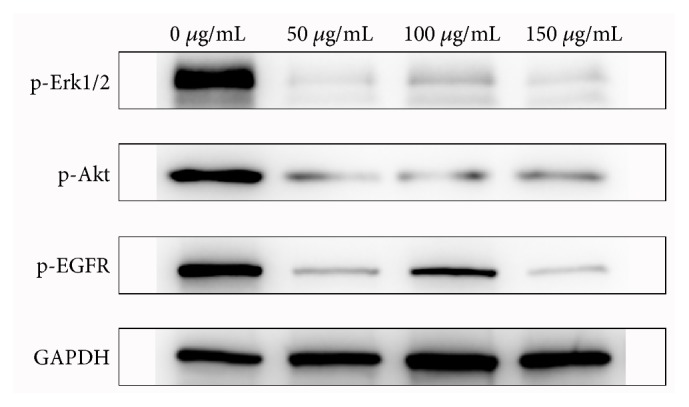
Analysis of the effect of Triphala on the EGFR signaling cascade. The Western blots compare lysates of untreated and Triphala-treated MGC803 cells probed with p-EGFR, p-ERK1/2, and p-AKT monoclonal antibodies as well as with an anti-GAPDH antibody as a control.

Functional activation of EGFR occurs in most human cancers. It plays an important role in regulating the proliferation and migration of cancer cells. To investigate the effect of Triphala on EGFR signaling pathways, Western blotting experiments were employed to determine the levels of phosphorylated EGFR signaling factors. A significant decrease in the levels of p-EGFR, p-Akt, and p-ERK1/2 was observed after the treatment with different concentrations of Triphala (Figure 3). This finding suggested that the anticancer mechanism of Triphala may involve the downregulation of the EGFR/Akt/ERK signaling pathway.

## 4. Discussion

Despite recent advances in diagnosis and treatment, gastric cancer is still a global health challenge with a high rate of morbidity and mortality, and a notoriously poor prognosis due to the malignant nature and limited treatment options. Complete surgical resection is the primary treatment for gastric cancer. Adjuvant radiotherapy and chemotherapy are often conducted after the operation. However, recurrence and metastasis still frequently occur and remain the major obstacles for long‐term survival [7]. Therefore, novel approaches such as immunotherapies have been introduced to improve the prognosis of patients [8, 9]. Meanwhile, traditional medicines have been also proven effective in increasing the overall survival of patients with gastric cancer [3]. In fact, many traditional medical systems developed therapeutic strategies for cancers, although these experience-based medicines are not always evidence-based.

In Tibetan medical theories, tumor or* ‘Bras Nad* in Tibetan is caused by the dysregulation of* Nyipa gSum* (three bodily humors), while Triphala is capable of rebalancing the three humors and, therefore, could be potentially used as a therapeutic in various of cancers [5, 10]. In Tibetan medical practices, Triphala not only is used for the treatment of gastric cancer, but is also prescribed as a basic recipe to make many famous formulations, such as* Rin Chen sBrang sByor*,* Rin Chen Mang sByor*,* bTso bKru zLa Shel, *etc. However, there was no experimental or clinical evidence on which to support that Triphala affects the progression of gastric cancer. In this study, we examined the antimalignancy and antimetastasis effects of Triphala on the human gastric cancer cell line MGC-803. Cell viability results from the CCK-8 assay showed that Triphala significantly suppressed the proliferation of MGC-803 cells* in vitro*. Accordingly, a reduction in the population of xenografted tumor cells was observed in the manipulated zebrafish. Furthermore, the results of the Transwell migration assay illustrated that Triphala could significantly reduce the migratory capability of MGC-803 cells. The* in vivo* study further demonstrated that Triphala is able to inhibit the metastasis of tumor cells from the primary lesion to distant sites. Therefore, the data suggested Triphala is a promising anticancer agent with efficacy against the growth and metastasis of gastric cancer cells* in vitro* and* in vivo*.

In addition to the anticancer properties, cumulative evidence has been provided that Triphala has a wide range of biological activities from the direct elimination of pathogenic microorganisms to the modulation of immune responses in the host. There is a consensus that untreated* Helicobacter pylori* infections and lingering mucosal inflammations are the main factors for the development of gastric cancer [11], but, interestingly, Triphala has shown efficacy against* H. pylori in vitro* and peptic ulcer disease in rodent models [12–14]. Furthermore, studies have revealed that Triphala exhibits significant radio- and chemoprotective activities in both cellular and* in vivo* model [15, 16].* In vitro* assessment and a study in the fruit fly model indicate that Triphala may affect the gastrointestinal flora by promoting the replication of beneficial bacteria while inhibiting the growth of pathogenic species [17, 18]. Hence, the clinical application of Triphala could be beneficial to decrease the number of new cases of gastric cancer and increase the survival rate and quality of life in patients with gastric cancer.

As described in the introduction, Triphala is comprised of three edible fruits; thus, its safety properties should be relatively superior to common chemotherapeutic agents. In our previous study, the half-lethal dose (LD_50_) of Triphala for zebrafish was found to be 542.44±56.35 *μ*g/mL, indicating that it appears to be safe to use Triphala as an adjunctive and alternative medicine.

However, it must be pointed out that this research was mostly performed on the cellular level. Triphala, as a multicomponent therapeutic is predicted to have multiple targets and functions. The observed antitumor activity of Triphala is supposed to be a synergistic effect caused by the regulation of multiple signaling pathways. Although the anticancer mechanism was found to be associated with the EGFR/Akt/ERK signaling pathway, further studies on the molecular level are still required to fully discover the underlying mechanism of Triphala. In addition, only one cell line was included in this study; therefore, the further analysis using additional gastric cancer cell lines is needed for validation. Furthermore, only a limited* in vivo* experiment was included in this project, therefore further confirmatory experiments as well as more in-depth studies are still needed to better understand of the pharmacological and toxicological properties of Triphala against human gastric cancer.

In conclusion, to our knowledge, this study is the first to provide evidence for the anticancer effect of Triphala against gastric cancer. It indicated that Triphala is a promising anticancer agent with potent proliferative and metastatic inhibitory effects on gastric cancer cells* in vitro* and* in vivo*. However, the complex pharmacological and toxicological characteristics and the underlying mechanism will be further assessed in future studies.

## Data Availability

The data used to support the findings of this study are included within the article.

## Abbreviations

CCK: Cell counting kit
dpf: Days postfertilization
IC_50_: Half maximal inhibition concentration
LD_50_: Half-lethal dose
MFI: Mean fluorescence intensity
OD: Optical density
PI: Propidium Iodide.
